# Potential Immunotoxic Effect of Thimerosal: Compound Alters Dendritic Cell Response *in Vitro*

**Published:** 2006-07

**Authors:** Julia R. Barrett

Thimerosal, an ethylmercury-based compound used for decades as a vaccine preservative, has previously been linked to neurotoxic effects. New research reveals that it may also affect the immune system by altering how dendritic cells respond to biochemical signals **[*EHP* 114:1083–1091; Goth et al.]**.

Dendritic cells are influential primary actors in the immune system’s response to infectious invasion of the body. Once activated, a single dendritic cell can direct hundreds of T cells against an infectious agent. This ability, however, depends on the dendritic cell responding appropriately to signals.

Previous studies by other researchers have indicated that thimerosal is an immunotoxicant, but its specific targets were unknown. Hypothesizing that dendritic cells might be sensitive targets, the researchers cultured bone marrow–derived dendritic cells from mice and assayed how both mature and immature cells responded to activation following treatment with thimerosal. They especially focused on the responses of inositol 1,4,5-trisphosphate and ryanodine receptors (IP_3_R and RyR, respectively), which are known thimerosal targets. These gatekeepers of intracellular calcium stores are essential for signaling activities affecting dendritic cell function and maturation.

The team showed for the first time that both mature and immature dendritic cells express isoforms of these receptors, IP_3_R1 and RyR1. Upon activation with the cellular energy source adenosine triphosphate, immature control cells responded with a measurable rise and fall in intracellular calcium concentration that involved RyR1 building upon the initial IP_3_R1-controlled calcium release and afterward working with IP_3_R1 to bring calcium down to resting levels.

Exposure to thimerosal at concentrations as low as 20 ppb altered the time course of these responses, however, and prolonged the length of time that intracellular calcium levels remained elevated. One possible consequence of these sustained calcium levels is a change in the rate and timing of dendritic cells’ secretion of interleukin-6, a chemical that triggers further immune system action. Exposure to thimerosal at concentrations above 200 ppb caused immature dendritic cells to die.

The continuing use of thimerosal in some vaccines and other products warrants further investigation of possible immunotoxic effects of this compound and its constituent ethylmercury. The researchers also note that the human *RyR1* gene is highly polymorphic, an observation that raises several questions about the role of RyR1 in the immune system’s genetic vulnerability to mercury.

